# Identification of retinoblastoma binding protein 7 (Rbbp7) as a mediator against tau acetylation and subsequent neuronal loss in Alzheimer’s disease and related tauopathies

**DOI:** 10.1007/s00401-021-02323-1

**Published:** 2021-05-12

**Authors:** Nikhil Dave, Austin S. Vural, Ignazio S. Piras, Wendy Winslow, Likith Surendra, Joanna K. Winstone, Thomas G. Beach, Matthew J. Huentelman, Ramon Velazquez

**Affiliations:** 1 Arizona State University-Banner Neurodegenerative Disease Research Center at the Biodesign Institute, Arizona State University, Tempe, AZ USA; 2 Neurogenomics Division, Translational Genomics Research Institute, Phoenix, AZ USA; 3 Arizona Alzheimer’s Consortium, Phoenix, AZ USA; 4 School of Life Sciences, Arizona State University, Tempe, AZ USA; 5 Civin Laboratory for Neuropathology, Banner Sun Health Research Institute, Sun City, AZ USA

**Keywords:** Alzheimer’s, Tauopathies, Rbbp7, PS19, Acetylation, Neuronal loss

## Abstract

Evidence indicates that tau hyper-phosphorylation and subsequent neurofibrillary tangle formation contribute to the extensive neuronal death in Alzheimer’s disease (AD) and related tauopathies. Recent work has identified that increased tau acetylation can promote tau phosphorylation. Tau acetylation occurs at lysine 280 resulting from increased expression of the lysine acetyltransferase p300. The exact upstream mechanisms mediating p300 expression remain elusive. Additional work highlights the role of the epigenome in tau pathogenesis, suggesting that dysregulation of epigenetic proteins may contribute to acetylation and hyper-phosphorylation of tau. Here, we identify and focus on the histone-binding subunit of the Nucleosome Remodeling and Deacetylase (NuRD) complex: Retinoblastoma-Binding Protein 7 (Rbbp7). Rbbp7 chaperones chromatin remodeling proteins to their nuclear histone substrates, including histone acetylases and deacetylases. Notably, Rbbp7 binds to p300, suggesting that it may play a role in modulating tau acetylation. We interrogated Rbbp7 in post-mortem brain tissue, cell lines and mouse models of AD. We found reduced Rbbp7 mRNA expression in AD cases, a significant negative correlation with CERAD (neuritic plaque density) and Braak Staging (pathogenic tau inclusions) and a significant positive correlation with post-mortem brain weight. We also found a neuron-specific downregulation of Rbbp7 mRNA in AD patients. Rbbp7 protein levels were significantly decreased in 3xTg-AD and PS19 mice compared to NonTg, but no decreases were found in APP/PS1 mice that lack tau pathology. In vitro, Rbbp7 overexpression rescued TauP301L-induced cytotoxicity in immortalized hippocampal cells and primary cortical neurons. In vivo, hippocampal Rbbp7 overexpression rescued neuronal death in the CA1 of PS19 mice. Mechanistically, we found that increased Rbbp7 reduced p300 levels, tau acetylation at lysine 280 and tau phosphorylation at AT8 and AT100 sites. Collectively, these data identify a novel role of Rbbp7, protecting against tau-related pathologies, and highlight its potential as a therapeutic target in AD and related tauopathies.

**Supplementary Information:**

The online version contains supplementary material available at 10.1007/s00401-021-02323-1.

## Introduction

Alzheimer’s disease (AD) is the most common neurogenerative disorder leading to dementia [3]. Clinically, AD is characterized by impairments in cognition, deficits in developing new memories, and a loss of long-term memories as the disease progresses [6, 22]. The hallmark neuropatholgies of AD include extracellular plaques composed predominately of the amyloid‐β (Aβ) peptide, intra-neuronal tangles of hyper-phosphorylated tau, and synaptic and neuronal loss [5, 22]. Currently, more than 5 million Americans are affected in the United States, and this number is expected to reach over 14 million by 2050 [3]. There are no approved clinical therapies to halt the progression or treat individuals in advanced stages of AD, necessitating the need for more research to identify novel therapeutic targets and amelioration strategies [3].

Pathological tau protein has been shown as a key player in neuronal death in AD and a related class of neurodegenerative diseases known as tauopathies [18, 27, 44]. In its normal function, tau is a scaffolding protein that stabilizes microtubules [50]. In tauopathies like AD, Frontotemporal dementia, Corticobasal degeneration, and Pick’s disease, tau is hyper-phosphorylated, reducing its binding affinity to microtubules and resulting in accumulation into insoluble intra-neuronal tau filaments [20, 21, 28]. Numerous reports have shown that various phosphorylation sites of tau, in particular phosphorylation at Ser202/Thr205 (AT8) and Ser212/Thr214 (AT100), are highly associated with increased intra-neuronal filaments and neuronal loss [1, 4]. Researchers have identified the sequential appearance of specific tau phospho-dependent epitopes, revealing that AT100 phosphorylation appears after AT8 in human AD post-mortem brain tissue [33]. Braak Staging, a well-established method that scores the accumulation of phospho-tau, is based on AT8 antibody staining [11, 12]. These findings are consistent with work in mouse models with human tau mutations [1]. The PS19 mouse model, which harbors the mutant human tau gene (TauP301S) that causes familial frontotemporal dementia [52], shows significant neuronal loss and a prominent AT8 staining compared to other tau phosphorylation epitopes [1, 52]. While there is ample evidence that tau hyper-phosphorylation leads to neurofibrillary tangle (NFT) development and neuronal loss, the complete mechanisms underlying tau pathogenesis remain largely elusive [18, 26, 27, 30, 56].

More recently, work has highlighted the role of tau acetylation in the pathological accumulation of tau [15, 32, 34, 35]. In particular, the lysine 280 within microtubules is the major binding site for tau acetylation [15]. Upon acetylation, there is an impairment in tau-microtubule interactions that promotes the aggregation of pathological tau [15]. Indeed, tau acetylation is now considered a mechanism by which normal soluble tau becomes disengaged from the microtubules, thereby forming tau inclusion [15]. Tau acetylation has been observed in the brains of patients with AD and is known to precede the accumulation of NFTs, suggesting that tau acetylation is an early event in tau-mediated neurodegeneration [34, 35]. The lysine acetyltransferase p300 has been shown to be aberrantly activated in tauopathies, directly acetylating tau at lysine 280 [14]. Reports have found that p300 increases the acetylation of tau and thereby promotes its aggregation and accumulation [15, 34, 35]. Inhibition of p300 has been shown to reduce tau accumulation, tau pathology and cognitive deficits in the PS19 mouse model of tauopathies [34]. However, what upstream proteins may regulate p300 and the progression of tau acetylation remain to be known.

Recent work has highlighted the emerging role of the epigenome in tau pathogenesis, suggesting that dysregulation of epigenetic proteins may contribute to acetylation and hyper-phosphorylation of cytoplasmic tau [53]. The epigenome is responsible for the dynamic regulation of protein expression largely through molecular modification of chromatin [2]. There are several epigenetic complexes which have been implicated in epigenomic dysfunction related to AD [31, 42]. Here, we focus on a histone-binding subunit of the Nucleosome Remodeling and Deacetylase (NuRD) complex: Retinoblastoma-Binding Protein 7 (Rbbp7). Rbbp7 is responsible for chaperoning chromatin remodeling proteins to their nuclear histone substrates, including histone acetylases (HATs) and histone deacetylases (HDACs) [13, 54, 55]. Rbbp7 modulates the epigenetic activity of these chromatin remodeling proteins, thereby modulating expression of target genes [13, 54]. Interestingly, Rbbp7 binds to p300 [54]. Given this protein interaction, Rbbp7 may play a vital role in regulating p300-induced tau acetylation and subsequent tau pathogenesis in AD and related tauopathies.

While Rbbp7 has been identified to play a role in chaperoning chromatin remodeling proteins, it has yet to be determined whether it plays a direct role in AD and related tauopathies [54]. Here, we interrogate Rbbp7 in post-mortem human brain tissue of patients with AD versus age-matched controls, immortalized hippocampal cells and primary cortical neuron lines, and modulate its expression in the PS19 mouse model of tauopathy. We hypothesize that Rbbp7 serves as an upstream mediator of tau pathogenesis whose expression reduces the acetylation of tau.

## Methods

### Human brain samples and mice

**Fig. 1:**
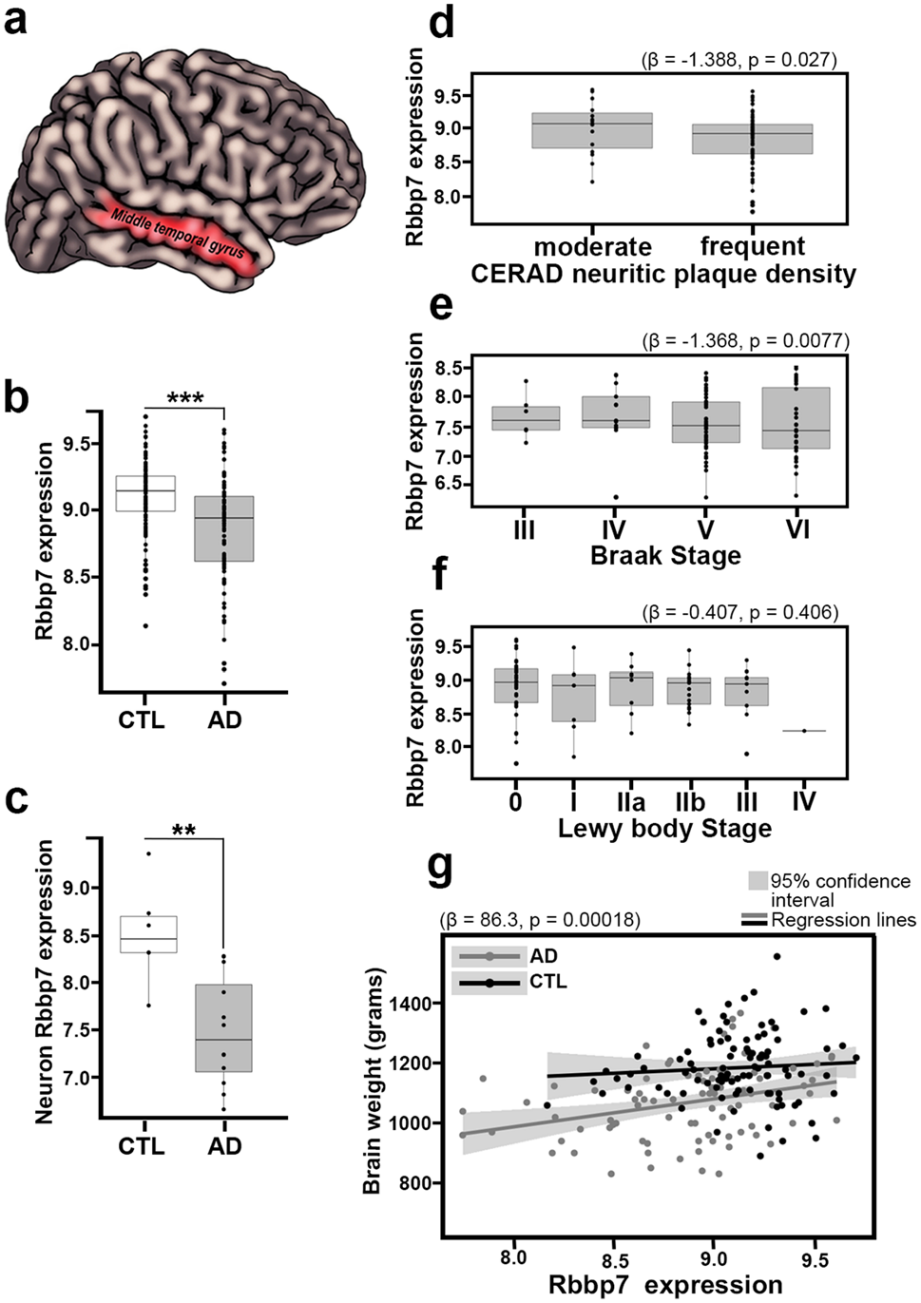
Rbbp7 expression is decreased in AD brain tissue and negatively correlate with CERAD neuritic plaque density and Braak Stage. **a**–**c** AD patients show a significantly lower Rbbp7 expression in the middle temporal gyrus (MTG) that is neuronal specific compared to age-matched CTL. **d**–**f** AD patients show a significant negative correlation between Rbbp7 mRNA expression and both CERAD neuritic plaque density and Braak Stage, but no correlation with Lewy body stage. **g** Linear regression analysis reveals that as Rbbp7 expression goes down, so does brain weight. Box plots: center line represents the median, the limits represent the 25th and 75th percentile, and the whiskers represent the minimum and maximum values of the distribution. **p < 0.01, ***p < 0.001

Brain samples used included the middle temporal gyrus (MTG; Broadmann area 21; Fig. 1a) obtained from the Arizona Study of Aging and Neurodegenerative Disorders/Brain and Body Donation Program as previously described [7, 39]. A total of 187 brain samples (*n* = 89 AD; *n*  = 98 age-matched controls; CTL) were used for the analysis of Rbbp7 mRNA expression by microarray and neuropathological hallmarks including CERAD neuritic plaque density, Braak neurofibrillary staging, Lewy body stage, and brain weight correlations as previously described [7, 39]. The complete microarray dataset is available at the Gene Expression Omnibus repository (GSE132903). AD and CTL subjects were not significantly different in age or sex distribution. The age at autopsy for AD patients was 84.66 ± 6.72 years (range: 70–98 years) and for CTL was 84.98 ± 6.90 years (range: 70–102). The mean post-mortem autopsy intervals (PMI) were 2.79 and 3.12 h for AD and CTL cases, not significantly different. The neuropathological characteristics of subjects are given in Table 1. AD was defined as meeting the National Institute on Aging-Reagan Institute (NIA-RI) “intermediate” or “high” criteria, with no concurrent defined neurodegenerative or cerebrovascular conditions, while the CTL category was defined as being NIA-RI “low” “intermediate” or “criteria not met” and without dementia or parkinsonism [57]. National Institute on Aging—Alzheimer’s Association criteria were not used because all of the subjects were autopsied before these newer criteria were published [23]. Some of the subjects had concurrent Lewy body disease and/or TDP-43 proteinopathy (Table 1), but not meeting diagnostic criteria for either dementia with Lewy bodies (DLB) or frontotemporal lobar degeneration with TDP-43. The AD cases with TDP-43 proteinopathy all are consistent with the newly defined entity, limbic-predominant age-related TDP-43 encephalopathy (LATE) [37]. We used MTG brain tissue because it is a standard region assessed for the NIA-RI classification system and is the most-affected neocortical area assessed by NIA-RI [57].

**Table 1:** Neuropathological variables for Alzheimer’s disease (AD) and control (CTL) cases

Braak staging							
Status (*n*)	I	II	III	IV	V	VI	*p*
AD (89)	0 (0.0)	0 (0.0)	6 (6.7)	13 (14.6)	42 (47.2)	28 (31.5)	< 2.2E−16
CTL (97)	17 (17.5)	22 (22.7)	37 (38.1)	21 (21.6)	0 (0.0)	0 (0.0)	

CERAD neuritic plaque density							
	Zero	Sparse	Moderate	Frequent	*p*		
AD (89)	0 (0.0)	0 (0.0)	15 (16.9)	74 (83.1)	< 2.2E−16		
CTL (98)	34 (34.7)	31 (31.6)	26 (26.5)	7 (7.1)			

Brain weight (g)							
Status (*n*)	Mean	*s*	Range	p*			
AD (88)	1069.66	121.7	830–1370	3.4E−13			
CTL (98)	1186.43	114.0	890–1560				

MMSE							
Status (*n*)	Mean	*s*	Range	p**			
AD (57)	11.79	9.11	0–28	< 2.2E−16			
CTL (51)	28.12	1.76	24–30				

NIA-Reagan neuropathological AD classification							
Status (*n*)	Not AD	Low	Intermediate	High	*p*		
AD (89)	0 (0.0)	0 (0.0)	19 (21.3)	70 (78.7)	< 2.2E−16		
CTL (98)	34 (34.7)	35 (35.7)	29 (29.6)	0 (0.0)			

Unified staging system for Lewy body disorders							
Status (*n*)	0	I	IIa	IIb	III	IV	*p*
AD (85)	43 (50.6)	8 (9.4)	8 (0.094)	17 (0.200)	9 (0.106)	1 (0.012)	8.7E−05
CTL (77)	61 (79.2)	3 (3.9)	8 (10.4)	1 (1.3)	3 (3.9)	1 (1.3)	

Density score for amygdala TDP-43 pathology							
Status (*n*)	0	Sparse	Moderate	Frequent	*p*		
AD (55)	35 (63.6)	6 (10.9)	5 (9.1)	9 (16.4)	4.3E−01		
CTL (38)	29 (76.3)	4 (10.5)	3 (7.9)	2 (5.3)			

Post-mortem brain tissue (bulk tissue) of the Middle Temporal Gyrus were characterized with RNA profiling microarrys (AD = 89; CTL = 98). Categorical variables distribution was compared using Fisher's Exact test

*Linear regression adjusted by sex

**Wilcoxon test

Three transgenic lines of AD mice were utilized. The non-transgenic (NonTg) and 3xTg-AD mice were generated as described previously [38]. 3xTg-AD mice express a Swedish mutation of the amyloid precursor protein (APP), a mutated presenilin (PS1 M146V) to accelerate amyloid deposition, and a mutated human tau (P301L), leading to tau pathology. Our colony of 3xTg-AD male mice display large pathological variability whereas females exhibit consistent neuropathology and cognitive deficits that are predictable based on age [9]. Therefore, only female 3xTg-AD mice were used in this study. The generation of the APP/PS1 mice has been described previously [24]. The APP/PS1 mice are hemizygous for the amyloid precursor protein (APP) Swedish mutations (KM670/671Nl) and presenilin1 (PS1) deltaE9 mutation. We have backcrossed the APP/PS1 mice for 12 generations into a pure 129/SvJ background [46, 48]. Littermates served as the NonTg controls. The PS19 human tau transgenic mice and littermate controls were purchased from Jackson laboratories (Stock No: # 008169). The PS19 mice overexpress mutant human tau (TauP301S) which in humans causes familial frontotemporal dementia [52]. PS19 mice display the identified neuropathological hallmarks of human tauopathies including pathological behavioral phenotypes and neuronal death [52]. The APP/PS1, PS19 and their respective NonTg mice were balanced for sex. At four to five per cage, mice were kept on a 12-h light/dark cycle at 23 °C. Additionally, mice were allowed ad libitum access to food and water. All animal procedures were approved in advance by the Institutional Animal Care and Use Committee of Arizona State University.

### RNA extraction and microarray analysis

RNA extraction and microarray analysis were performed as previously described [39]. RNA was extracted from 60 mg of frozen MTG tissue using RNEasy Mini Kit (Qiagen). Samples were prepared and hybridized, and the raw data were background corrected, normalized and analyzed for outliers/low-quality samples as previously described [25, 39]. Briefly, the expression dataset was then annotated using lumiHumanAll.db, an R package [17]. After low expressed and non-annotated probes were excluded, the final dataset consisted of 26,583 probes corresponding to 21,122 genes. Additionally, if multiple probes matched to a single gene, summarization of data was not performed to prevent loss of information as the multiple probes can correspond to different isoforms/variants of the same gene. Variance of raw data was stabilized (VST) and normalized as previously described [39]. Moderated t-statistics were used to detect differences in expression between AD and CTL adjusting for sex, expired age, PMI and RNA integrity number (RIN), and FDR *p* value correction method was used to account for multiple testing. FDR less than 0.05 was considered statistically significant. Differential expression analysis and VST were conducted using the R package Lumi [17].

We further reanalyzed the publicly available dataset GSE5281 derived from laser-captured micro-dissected neurons of tissue obtained from tissue banks at Banner Sun Health Research Institute and Washington University [29]. To create a completely independent cohort, samples form Banner Sun Health Research were excluded from the analysis. This independent dataset was processed and analyzed, and differential expression was computed as previous described [39]. *p* values were corrected for multiple testing using the FDR method. FDR less than 0.05 was considered statistically significant.

### Protein extraction for Western Blot

Mice were euthanized and had their hippocampus dissected and flash frozen. Flash-frozen tissue was homogenized in a T-PER tissue protein extraction reagent, and supplemented with protease (Roche Applied Science, IN, USA) and phosphatase inhibitors (Millipore, MA, USA). The homogenized tissues were centrifuged at 4 °C for 30 min. The supernatant (soluble fraction) was stored at − 80 °C. Western blotting was performed as previously described [49]. Protein extract was denatured and run in precast Novex gels (Life Technologies). Proteins were then transferred to nitrocellulose membranes using an iBlot (Life Technologies). Membranes were then blocked in 5% powdered milk in Tris-buffered saline with Tween (TBST). Primary antibodies were incubated in 5% milk in TBST overnight. Membranes were washed in TBST the following day and then incubated in fluorescent secondary antibody. The membranes were then washed again, and imaged and quantified using a LI-COR Odyssey CLx (LI-COR Biosciences) and Image Studio (version 1.0.11, LI-COR Biosciences).

### In vitro experiments

For cell viability assessment in HT-22 cells, a total of 12 independent wells were seated with cells and treated with the various plasmids. Plasmids used in these experiments (pcDNA3-EGFP, pRK5-EGFP-Tau P301L, and P2RP3-hRbbp7) were transfected into HT-22 cells as previously described [51]. Three wells were assigned to one of the four groups: (1) pcDNA3-EGFP (control), (2) P2RP3-hRbbp7, (3) pcDNA3-EGFP, pRK5-EGFP-Tau P301L (co-transfection with GFP-TauP301L) and (4) pcDNA3-EGFP, pRK5-EGFP-Tau P301L, and P2RP3-hRbbp7 (GFP-TauP301L-hRbbp7). 30 h after transfection, MTT (MTT 3-(4,5-dimethylthiazol-2-yl)-2,5-diphenyltetrazolium bromide) assay was used to assess cell death. Samples were tested in triplicate. All absorbance values were normalized to the control group, HT-22 cells transfected with pcDNA3-EGFP.

For cell viability assessment in primary cortical neurons, cells were harvested from newborn C57BL/6 pups, plated into 6-well dishes and cultured 48 h prior to transfection using the Primary Neuron Isolation Kit from Pierce (Pierce Cat# 88280). Plasmids used in these experiments (pcDNA3-EGFP, pRK5-EGFP-Tau P301L, and P2RP3-hRbbp7) were transfected into the primary neuron cultures as previously described [51]. 30 h after transfection, a MTT (MTT 3-(4,5-dimethylthiazol-2-yl)-2,5-diphenyltetrazolium bromide) assay was used to assess cell death. Samples were tested in triplicate. All absorbance values were normalized to the control group, primary neurons transfected with pcDNA3-EGFP.

For cytotoxicity assessment, primary cortical neurons were harvested from newborn C57BL/6 pups, plated into 24-well plates in quadruplicate for each test group and cultured 48 h prior to transfection using the Primary Neuron Isolation Kit from Pierce (Pierce Cat# 88280). Plasmids used in these experiments (pcDNA3-EGFP and P2RP3-hRbbp7) were transfected into the primary neuron cultures as previously described [51]. 30 h after transfection, 0.1 μg/mL human TNFα (PeproTech Cat# 300-01A) was added to the treatment groups and incubated for 4 h to induce cell death. Cytotoxicity was measured by lactate dehydrogenase release using Promega’s CytoTox 96^®^ Non-Radioactive Cytotoxicity Assay (Promega Cat# G1780). Samples were tested in quadruplicate. All absorbance values were normalized to the control group, primary neurons transfected with pcDNA3-EGFP.

### Viral construct generation for in vivo experiments

Adeno-associated virus (AAV) vectors were generated from Vector biolabs: AAV2-CamKIIa-Rbbp7-T2A-GFP-WPRE (abbreviated AAV-Rbbp7) and AAV2-CamKIIa-GFP-WPRE (abbreviated AAV-GFP). The experimental virus was generated to target the Rbbp7 gene. The CamKIIa promoter allows for neuron-specific expression of Rbbp7. T2A is a self-cleavage signal for co-expression of two proteins. WPRE is an enhancer for higher protein expression. GFP allows for detection of viral diffusion of the AAVs. Final viral titer was 7.6 × 10^12^ GC/ml. The sequence of the AAV-Rbbp7 was generated using the NCBI mouse genome sequence reference sequence number: BC003785.

### Stereotaxic surgeries

Both male and female 3-month-old PS19 and NonTg mice received one of the two AAVs into CA1 of the hippocampus using the following coordinates from bregma: − 2.1 mm anteroposterior; ± 1.8 mm lateral/medial; − 1.5 mm dorsoventral from the skull) as previously described [47]. A total of 2.5 μl/hemisphere of the pseudorandom assigned AAV was delivered at 0.5 μl/min after which the needle was left in place for an additional three minutes before being removed.

### Morris water maze testing

To assess spatial reference learning and memory, Morris water maze (MWM) testing was performed as previously described [46]. Each animal was given four trials per day for a total of 5 days. The location of the hidden platform remained in the same quadrant for all the animals; however, the start location was pseudorandomly selected. During the first 5 days of testing, each animal was given 60 s to locate the hidden platform. If the animal failed to reach the hidden platform in the 60 s, they were gently guided to its location. On the day six probe trial, the platform was removed, and the animals were given one 60 s trial to assess spatial reference memory. All trials were recorded with a video camera, and data were analyzed via the EthoVisionXT system from Noldus Information Technology.

### Tissue preparation for histology and image analysis

Mice were perfused with fresh 1× PBS and brains were extracted and subsequently fixed in a glass vial of 4% paraformaldehyde for 48 h. After fixing, a Leica VT1000 S vibratome was used to partition the tissue into 50 μm coronal sections and stored chronologically in a specimen plate with PBS containing 0.02% sodium azide. Coronal brain sections including the hippocampus underwent immunohistochemistry as previously described [46, 48] with the appropriate antibodies. Rbbp7 and AT8 images were taken at a resolution of *x* = 512, *y* = 512, and *z* = 1 using a 60× oil immersion objective and tau acetylation, p300 and GFP images were taken at a resolution of *x* = 1024, *y* = 1024, and *z* = 1 using a 40× oil immersion objective. A zoom of 1.5 was used and images were taken with the Leica DM2500 confocal microscope. Quantification was performed by taking signal area and intensity using Image J software. To quantify AT8- and AT100-positive cell counts, images from all animals were taken with a Zeiss Axio Imager A1 using a 5× objective. Images were photomerged to rebuild the image, and AT8- and AT100-positive cells numbers were obtained using imageJ. The experimenter was blinded to the group allocation.

### Antibodies

The following primary antibodies were used for tissue staining and western blot analysis: Acetyl Tau K280 (1:500 dilution, VWR, catalog #AS-56077), AT8 (1:1000 dilution, Thermo Fisher Scientific, catalog #MN1020), AT100 (1:1000 dilution, Thermo Fisher Scientific, catalog #MN1060), GFP (1:1000 dilution, Aves Labs, catalog #AB_2307313), NeuN (1:5000 dilution for chromogen staining, Abcam, catalog #024307P), NeuN (1:200 dilution for fluorescence staining, Millipore Sigma, #MAB377), p300 (1:100 dilution, Millipore Sigma, catalog #SAB4503485), Rbbp7 (1:1000 dilution, Abcam, catalog #ab3535).

### Unbiased stereology

NeuN-positive cells in the CA1 pyramidal layer of the hippocampus were quantified using the optical fractionator method as previously described [19]. All stereological analyses were performed by a single investigator that was blind to the groups. We sampled every sixth section throughout the rostrocaudal extent of the CA1 region. Sampling included both dorsal and ventral blades of the hippocampus. Stereoinvestigator 17-software (Micro-BrightField, Cochester, VT) was used to systematically sample throughout the designated region of interest. Counts were performed at predetermined intervals; Grid size (*X* and *Y* = 224 μm), and a counting frame (*X* and *Y* = 50 μm) and superimposed on the live image of the tissue sections. The sections were analyzed using a 63*x* × 1.4 PlanApo oil immersion objective. Sampling was optimized to produce a coefficient of error (CE) less than the observed biological variability. The Gunderson score remained less than or equal to 0.1. The thickness of each section was determined by focusing on the top of the section, zeroing the z-axis followed by focusing on the bottom of the section. The average tissue thickness was 23.3 μm with a range of 19.7 μm–25.7 μm. The dissector height was set at 15 μm, allowing for a 3-μm top guard zone and at least a 3-μm bottom guard zone. A total of 5–6 sections were evaluated per animal. Bright field photomicrographs were taken with the aid of a Zeiss Axio Imager.

NeuN antibody penetration throughout the depth of tissue sections was determined during the optical fractionator by visual analysis of immunolabeling throughout the *z*-axis and post-probe run examination of depth histograms which demonstrate marker placement in the *z*-axis [45]. NeuN antibody penetrated the full depth of the section, thus allowing for the equal probability of counting all objects, a prerequisite for unbiased stereology.

### Statistical analyses

Sample sizes used were similar to those reported in previously published papers [8, 46, 47]. The distribution of ordinal variables (CERAD neuritic plaque density, Braak stage, NIA-RI classification, Lewy body stage, and TDP-43 density score) between AD and CTL cases was analyzed using a Fisher’s Exact test. Brain weight between AD and CTL was compared by linear regression adjusting for sex, and MMSE was compared by Wilcoxon test. The correlation between CERAD neuritic plaque density and Rbbp7 mRNA expression was conducted by logistic regression. We conducted an ordinal logistic regression analysis to determine the presence of significant correlation with Rbbp7 mRNA expression and Braak stage and Lewy body stage, using mRNA expression as a predictor. The relationship between Rbbp7 mRNA expression and brain weight was conducted by linear regression adjusting for sex. For western blot, MTT and cytotoxicity assay, and tissue analysis, factorial Analysis of Variance (ANOVA) was performed followed by Bonferroni post hoc tests when appropriate. Repeated measured behavioral tasks were analyzed in SPSS using MANOVA syntax for a multivariate 3-way split plot design at alpha level *p* < 0.05. Syntax included omnibus tests, marginal means comparisons, and interaction contrasts for the Between subject x Between subject x Within Subject design followed by Bonferroni's post hoc tests, when appropriate. Linear correlations between GFP and p300 variables were calculated using the Pearson *r* analysis. A priori contrasts were conducted regardless of a significant genotype by AAV interaction: NonTg AAV-GFP vs. PS19 AAV-GFP; PS19 AAV-GFP vs. PS19 AAV-Rbbp7, and PS19 AAV-Rbbp7 vs. NonTg AAV-GFP. Examination of descriptive statistics revealed no other violation of any assumptions that required the use of statistical test other than the ones used. Significance was set at *p* < 0.05.

## Results

### Rbbp7 mRNA expression levels are decreased in post-mortem brain tissue of AD patients

To determine whether human brain tissue of patients with AD show dysregulated Rbbp7 levels, we first examined Rbbp7 mRNA expression in the middle temporal gyrus (MTG; Fig. 1a). We found significantly lower mRNA levels in AD patients (*n* = 89) compared to age and sex-matched controls (CTL) (*n* = 98; Log_2_ Fold Change = − 0.169, FDR adj-*p* = 0.0007; Fig. 1b). To determine whether Rbbp7 dysregulation was observed in neurons, we analyzed an independent mRNA dataset of AD (*n* = 6) and CTL (*n* = 12) patients from MTG laser-capture neurons. We found significantly lower neuronal Rbbp7 mRNA expression in AD patients compared to CTL (Log_2_ Fold Change = − 1.076, FDR adj-*p* = 0.0072; Fig. 1c). Next, we carried out regression analyses between Rbbp7 mRNA levels and various neuropathologies. Regression analyses of Rbbp7 mRNA revealed a significant inverse relationship to CERAD neuritic plaque density (Coefficient = − 1.388, *z* = 2.218, *p* = 0.027; Fig. 1d). Additionally, we found a significant inverse relationship with Braak Stage (Coefficient = − 1.368, *t* = − 2.663, *p* = 0.0077; Fig. 1e), a measure of pathological tau inclusions. Notably, we found no significant relationship between Rbbp7 mRNA and Lewy body Stage (Coefficient = − 0.407, *t* = − 0.830, *p* = 0.406; Fig. 1f). Lastly, we ran a linear regression and found a positive relationship illustrating that as Rbbp7 mRNA expression decreases, so does the brain weight of post-mortem brains (Coefficient = 86.3, *p* = 0.00018; Fig. 1g). Collectively, these data illustrate Rbbp7 mRNA dysregulation in AD brain tissue, identify neuronal specificity, and suggest a link between Rbbp7, AD neuropathology and brain weight.

### Rbbp7 is downregulated in AD mouse models with tau pathology

**Fig. 2:**
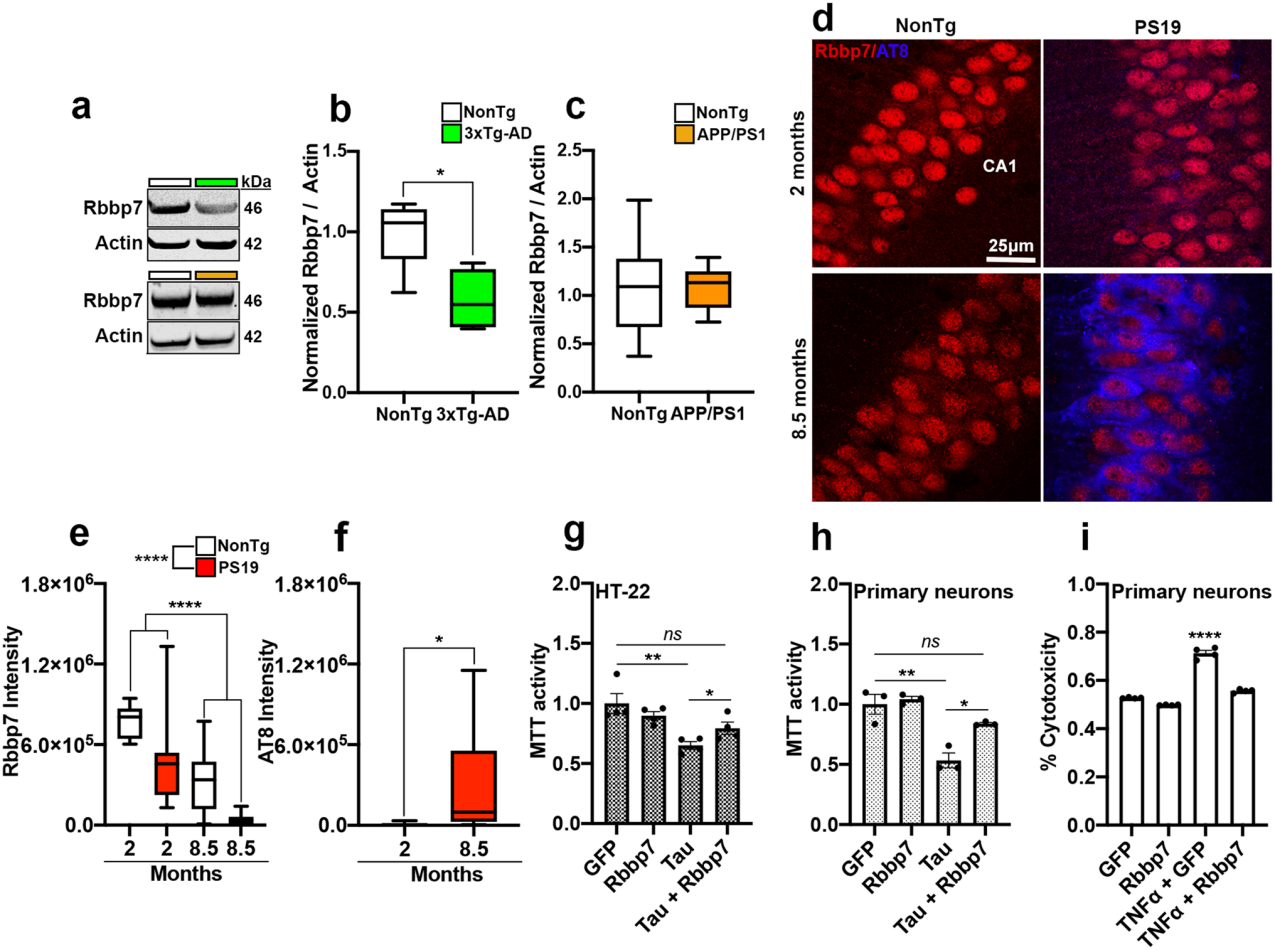
Rbbp7 protein levels are reduced in AD mouse model with tau pathology and increased expression protects against cell death in vitro. **a** Representative immunoblot of Rbbp7 and loading control β-actin. **b** 3xTg-AD mice have significantly decreased Rbbp7 protein levels compared to NonTg. **c** APP/PS1 mice show similar Rbbp7 levels compared to NonTg. **d** Hippocampal CA1 photomicrographs stained for Rbbp7 and AT8. **e** Quantitative analysis of Rbbp7 intensity reveals a significant reduction in PS19 mice over NonTg, and a signification reduction with age. **f** Quantitative analysis of AT8 intensity reveals a significant increase in 8.5-month-old PS19 mice over 2-month-old PS19 mice. **g**, **h** Overexpression of Rbbp7 significantly reduces TauP301L-induced cytotoxicity in HT-22 cells and primary cortical neurons. **i** Overexpression of Rbbp7 significantly reduces TNFα-induced cytotoxicity in primary cortical neurons. Box plots: center line represents the median, the limits represent the 25th and 75th percentile, and the whiskers represent the minimum and maximum values of the distribution. Scatter plots with bar graphs are means ± SE. *p < 0.05, **p < 0.01, ****p < 0.00001

To validate whether Rbbp7 is dysregulated in mouse models of AD, we first extracted the hippocampus of 12-month-old female 3xTg-AD (*n* = 4) and NonTg (*n* = 5) mice, which have well-documented cognitive decline and marked build-up of Aβ deposits and pathological tau at this age, and measured Rbbp7 levels via immunoblot [9]. We found significantly decreased Rbbp7 levels in 3xTg-AD mice compared to NonTg counterparts (*t*_(7)_ = 3.030, *p* = 0.0181; Fig. 2a, b). Next, we measured hippocampal Rbbp7 in both male and female APP/PS1 mice (*n* = 10) that does not possess any tau-related mutations, and littermate NonTg mice (*n* = 8) via immunoblot. APP/PS1 mice were 12 months old, an age where cognitive decline and Aβ deposits are well-documented [24, 46]. We found no significant differences in Rbbp7 levels between APP/PS1 and NonTg mice, illustrating an association between Rbbp7 and tau (Fig. 2a, c). Lastly, we examined Rbbp7 levels in the CA1 region of the hippocampus of PS19 mice, which show tau pathology and neuronal loss in this brain region but lack Aβ pathology [52]. Specifically, we stained tissue from two cohorts of PS19 mice and littermate NonTg controls (both male and female), one cohort was 2-month-old (*n* = 4 for NonTg; *n* = 5 for PS19) and the second was 8.5-month-old (*n* = 5 for NonTg; *n* = 8 for PS19) at sacrifice, against Rbbp7 and phosphorylated tau at Ser202/Thr205 (AT8; Fig. 2d). Notably, the 2 month cohort was selected as PS19 mice do not show tau pathology until 3 months of age [52]. We found a significant main effect of genotype for Rbbp7 intensity (*F*_(1,37)_ = 19.01, *p* < 0.0001; Fig. 2d, e), where PS19 mice show a significant reduction at the 2- and 8.5-month time points compared to NonTg counterparts. Additionally, we found a significant main effect of age (*F*_(1,37)=_44.27, *p *< 0.0001; Fig. 2d, e), where the 8.5-month mice show lower Rbbp7 intensity than the 2-month-old mice. For AT8 expression, we found a significant difference between the 2- and 8.5-month PS19 mice (*t*_(24)_ = 2.550, *p* = 0.0176; Fig. 2d, f), where the older mice had a significantly higher AT8 intensity than the younger mice. Collectively, these results identify a reduction of Rbbp7 in mouse models containing pathological tau, and illustrate that in PS19 mice, Rbbp7 is reduced prior to tau pathology and decreases significantly with advancing age.

### Rbbp7 overexpression rescues tau- and TNFα- induced cell death in HT-22 cells and primary cortical neurons

While we observed a correlation between tau pathology and Rbbp7 expression in both human brain tissue and mouse models of AD, the nature of this association is unclear. To elucidate the relationship between Rbbp7 and tau pathology, we first co-transfected an immortalized mouse hippocampal cell line (HT-22) with a plasmid expressing the familial AD mutant tau (TauP301L), as well as a human Rbbp7 (hRbbp7) plasmid, to assess cytotoxicity measured via MTT assay. At 30 h post transfection, our analysis showed a significant effect between cells transfected with control GFP, hRbbp7, and co-transfection with GFP-TauP301L, and GFP-TauP301L-hRbbp7 (*F*_(3,12)_ = 7.785, *p* = 0.0038; Fig. 2g). Post hoc analysis revealed no difference between GFP and Rbbp7 transfected cells (*p* = 0.2937). Co-transfection of GFP-TauP301L yielded a significantly higher cell death than the GFP transfection (*p* = 0.0056). Remarkably, cells co-transfected with GFP-TauP301L-hRbbp7 significantly reduced the rate of cell death compared to the GFP-TauP301L co-transfected group (*p* = 0.038), suggesting that Rbbp7 has a protective effect on TauP301L-mediated cell death. Furthermore, co-transfection of GFP-TauP301L-hRbbp7 did not significantly differ in cell viability when compared with control GFP transfection (*p* = 0.0762). Next, we tested this same paradigm in primary cortical neurons derived from C57BL/6 P0 mice. Our analysis showed a significant effect between cells transfected with control GFP, hRbbp7, and co-transfection with GFP-TauP301L, and GFP-TauP301L-hRbbp7 (*F*_(3,8)_ = 19.02, *p* = 0.0005; Fig. 2h). Post hoc analysis revealed that co-transfection of GFP-TauP301L yielded significantly higher cell death than the GFP transfection (*p* = 0.0015). Co-transfection with GFP-TauP301L-hRbbp7 significantly reduced the rate of cell death compared to the GFP-TauP301L co-transfected group (*p* = 0.0209), further illustrating that Rbbp7 has a rescue effect on TauP301L-mediated cell death. The co-transfection of GFP-TauP301L-hRbbp7 did not significantly differ in cell viability when compared with control GFP transfection (*p* = 0.3265). These data are consistent with the HT-22 immortalized cell results. Lastly, we were interested in determining whether Rbbp7 could reduce cytotoxicity independent of tau. We performed a cytotoxicity assay between GFP and hRbbp7 infected primary cortical neurons incubated with and without TNFα, an inducer of apoptotic cell death [40]. Our analysis showed a significant effect between cells incubated in TNFα and transfected with control GFP and hRbbp7 in % cytotoxicity (*F*_(3,12)_ = 243.1, *p* < 0.0001; Fig. 2i). Post hoc analysis revealed that transfection with hRbbp7 decreased TNFα induced cytotoxicity compared to GFP-transfected cells (*p* < 0.0001). Collectively, these results highlight both a protective role that Rbbp7 plays in tau-induced cell death and it’s potential to block TNFα-induced cell death independent of pathological tau.

### Rbbp7 overexpression rescues cell death in the PS19 mouse

**Fig. 3:**
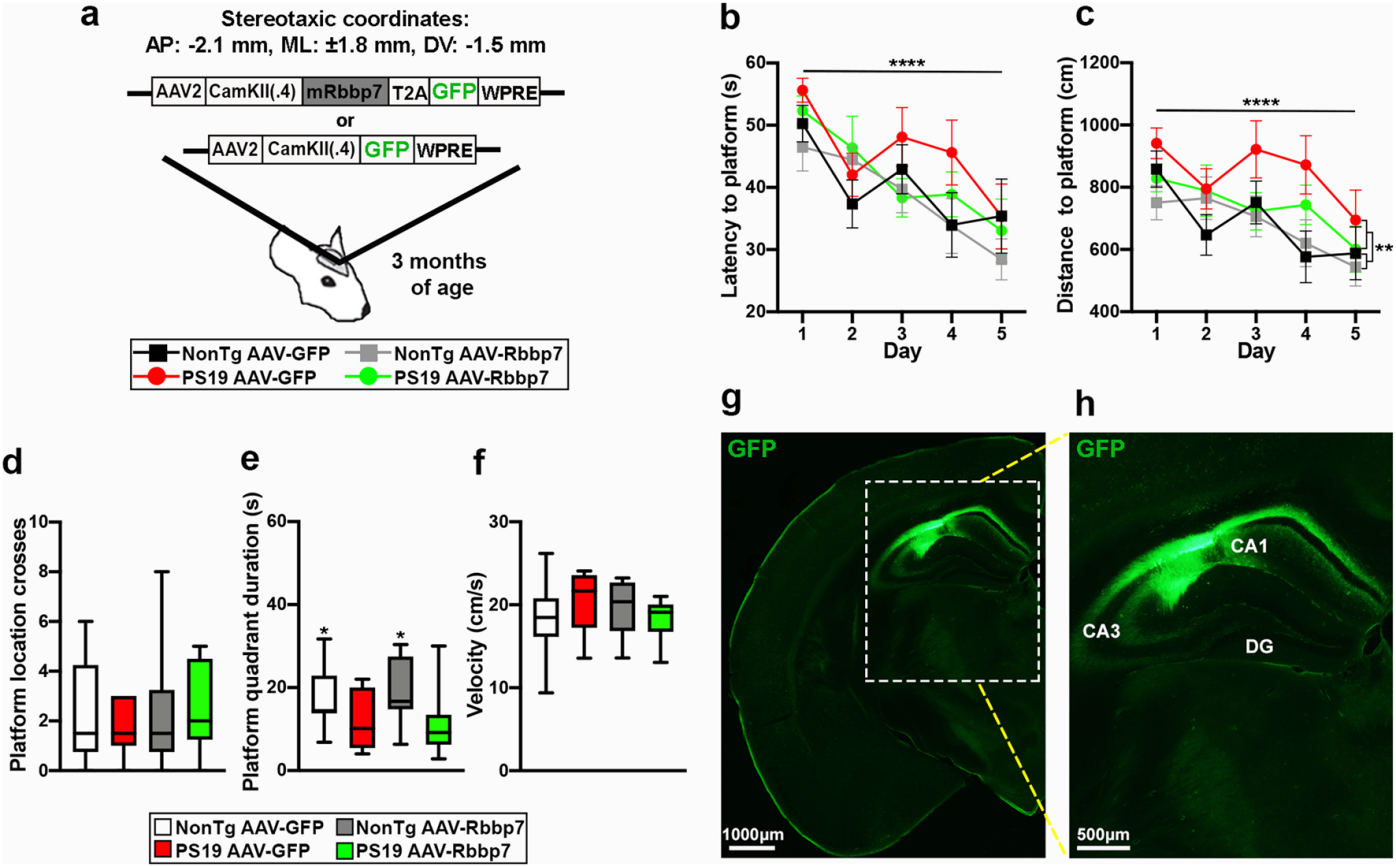
AAV-Rbbp7 into CA1 of the hippocampus does not improve spatial cognition. **a** The adeno-associated viruses (AAVs) bilaterally injected into CA1 of the hippocampus. [anterior–posterior (AP); medial–lateral (ML); dorsal–ventral (DV)]. **b**, **c** Escape latency and distance traveled in the 5-day learning phase of the Morris water maze. **d**–**f** Results of the day 6 probe trails of the MWM. **g**, **h** Low and high magnification photomicrographs depicting the spread of AAV with the GFP reporter in the hippocampus. Line graphs are means ± SE. Box plots: center line represents the median, the limits represent the 25th and 75th percentile, and the whiskers represent the minimum and maximum values of the distribution. ****p < 0.0001

Next, we sought to elucidate the relationship between Rbbp7 and tau in vivo. We injected 2.5 μl/hemisphere of the AAV overexpressing Rbbp7 (abbreviated AAV-Rbbp7) or the control AAV (abbreviated AAV-GFP), into CA1 of the hippocampus of 3-month-old PS19 (*n* = 9/AAV group) and littermate NonTg mice (*n* = 10/AAV group; Fig. 3a). Mice were aged to 7 months and tested in the MWM task. During the 5-day learning phase of the MWM, we found a significant main effect of day for latency (*F*_(4,132)_ = 12.34, *p* < 0.0001; Fig. 3b) and distance traveled (*F*_(4,132)_ = 7.03, *p* < 0.0001; Fig. 3c), illustrating all animals were capable of learning. We also found a significant main effect of genotype, where PS19 mice traveled significantly longer to find the platform than the NonTg mice (*F*_(1,33)_ = 7.81, *p* = 0.009, Fig. 3c). On the day six probe trial, the hidden platform was removed from the pool and the mice had 60 s to swim to assess spatial reference memory. We found no significant differences in the number of platform crosses in the platform location (Fig. 3d). We did find a significant main effect of genotype for platform quadrant duration, where the NonTg mice spent more time in the platform quadrant than the PS19 mice (*F*_(1,33)_ = 7.328, *p* = 0.017; Fig. 3e). Lastly, we found no significant differences in swim speed amongst the groups (Fig. 3f). Collectively, these results highlight a spatial cognition impairment in PS19 mice, and that the AAV-Rbbp7 was not capable of rescuing these deficits. We speculate that these results are likely due to the specific targeting of CA1 and no other regions associated with spatial cognition.

**Fig. 4:**
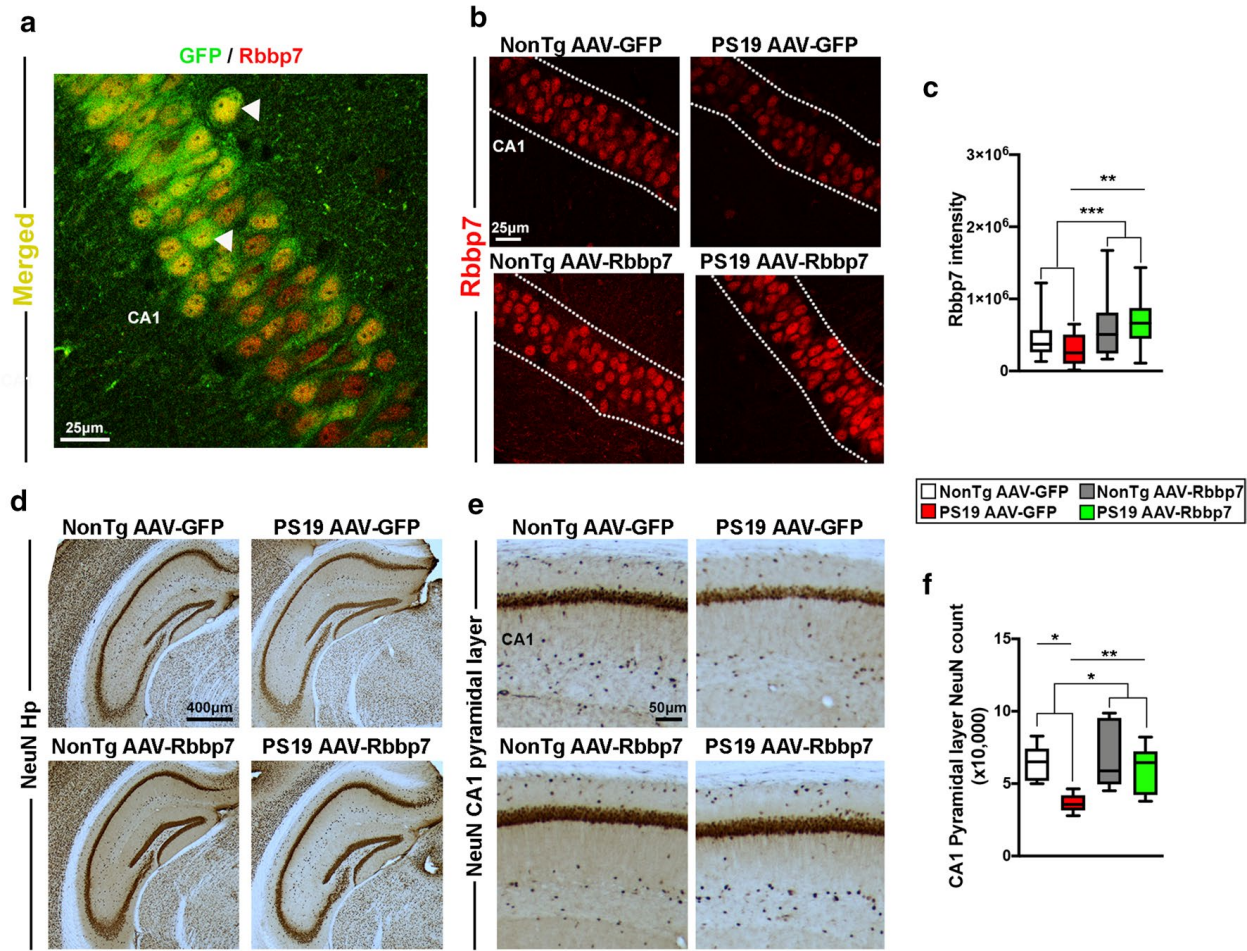
AAV-Rbbp7 injected into CA1 of the hippocampus increases Rbbp7 levels and reduces neuronal loss in PS19 mice. **a** High magnification photomicrograph depicting neurons labeled with the GFP reporter and Rbbp7. **b** Hippocampal CA1 photomicrographs stained for Rbbp7. Dotted lines depict CA1 area analyzed. **c** Quantitative analysis of Rbbp7 intensity reveals a significant increase in AVV-Rbbp7 injected mice. PS19 AAV-Rbbp7 have a higher expression of Rbbp7 than PS19 AAV-GFP mice. **d**, **e** Low and high magnification of hippocampal sections stained for NeuN. **f** Quantitative analysis of neuronal counts using unbiased stereology in the CA1 of the hippocampus. PS19 AAV-Rbbp7 mice show a significantly higher number of NeuN + cells compared to PS19 AAV-GFP and cell count number did not differ compared to NonTg mice. Box plots: center line represents the median, the limits represent the 25th and 75th percentile, and the whiskers represent the minimum and maximum values of the distribution. *p < 0.05, **p < 0.01. ***p < 0.001

All animals were subsequently euthanized and brains were prepared to examine the diffusion of the AAVs by immunostaining against GFP. We found that the AAV spread specifically in the CA1 region of the hippocampus (Fig. 3g, h). Additionally, we immunostained tissue for GFP and the neuronal marker NeuN to determine whether the AAVs selectively infected neurons. We found co-expression of the AAVs in neurons of the CA1 of the hippocampus (Supplementary Figure 1a, b). We immunostained sections for GFP and Rbbp7 to determine if our AAVs co-localized with our protein of interest and found co-staining in CA1 of the hippocampus (Fig. 4a). To determine whether the AAV-Rbbp7 altered Rbbp7 levels, we stained sections with an antibody against Rbbp7 and measured intensity in the CA1 region of the hippocampus. Quantitative analysis revealed a significant main effect of AAV, where the AAV-Rbbp7 injected mice had a significantly higher expression of Rbbp7 than the AAV-GFP injected counterparts (*F*_(1,70)_ = 12.10, *p* = 0.0009; Fig. 4b, c). A prior comparisons revealed that PS19 AAV-Rbbp7 mice had a significantly higher level of Rbbp7 intensity than the PS19 AAV-GFP mice (*p* = 0.0078). These result show that the AAV-Rbbp7 was able to infect neurons and increase levels of Rbbp7.

To determine whether overexpression of Rbbp7 rescues neuronal cell death, we stained sections with an antibody against the neuronal marker NeuN and performed unbiased stereology in the CA1 region of the hippocampus (Fig. 4d, e). PS19 mice begin to show cell death by 6.5 months of age [52]. Quantitative analysis of NeuN + cell counts revealed a significant main effect of genotype, where PS19 mice (*n* = 6 mice/AAV group) show a decreased number of neurons in CA1 compared to NonTg mice (*n* = 5 mice/AAV group) mice (*F*_(1,18)_ = 7.190, *p* = 0.0152; Fig. 4f). A prior comparisons revealed that PS19 AAV-GFP mice show a significant reduction in NeuN + cells compared to NonTg AAV-GFP mice (*p* = 0.0013). Notably, PS19 AAV-Rbbp7 mice show a significantly higher number of NeuN + cells compared to PS19 AAV-GFP (*p* = 0.0081), and the count number did not differ compared to NonTg mice (*p* = 0.7347). Collectively, these results show that Rbbp7 rescues cell death in the PS19 mouse model, which is consistent with the previously discussed in vitro cell experiments and the observed correlations between Rbbp7 mRNA and brain weight from humans.

### Rbbp7 overexpression reduces tau acetylation and phosphorylation in PS19 mice

**Fig. 5:**
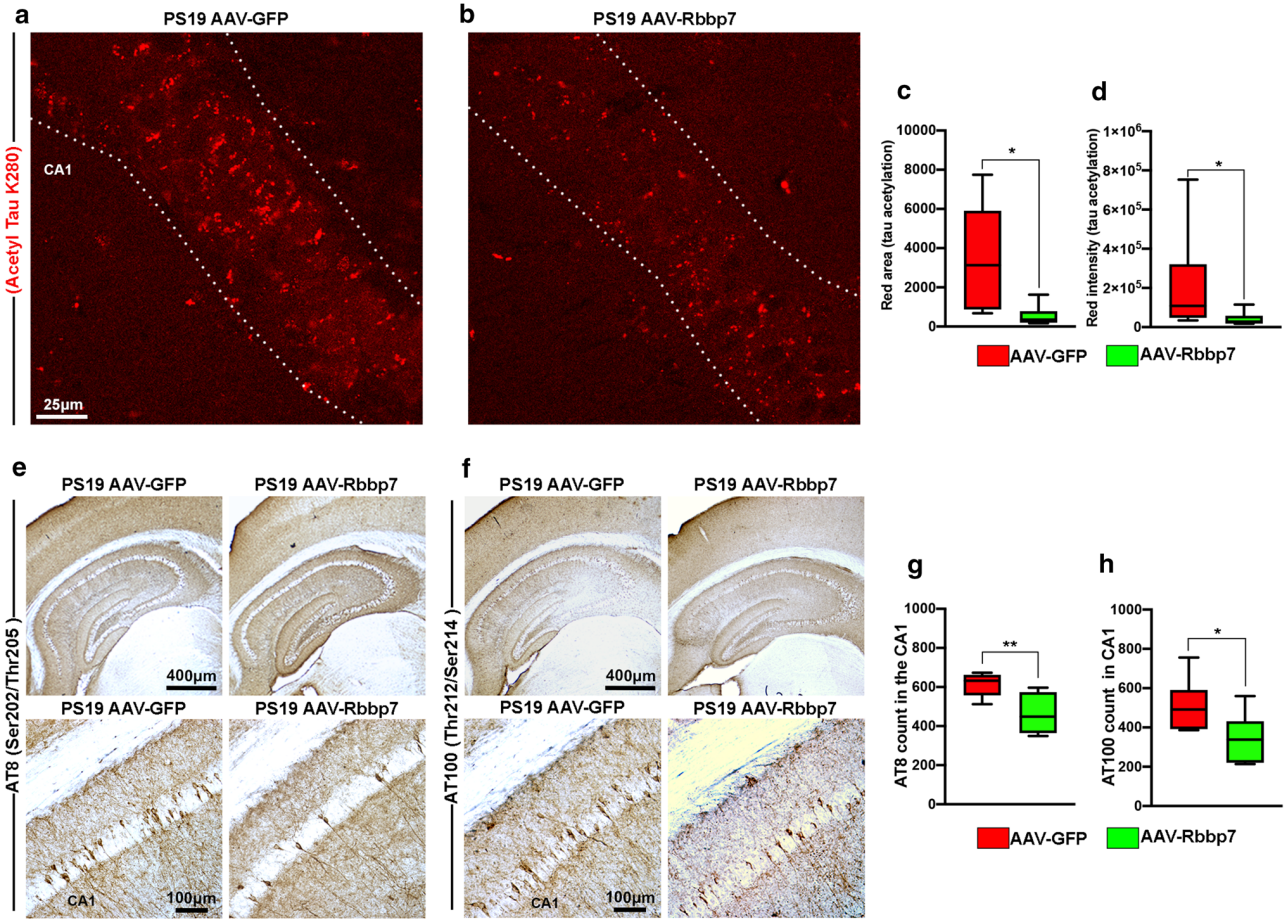
AAV-Rbbp7 reduces tau acetylation and phosphorylation in PS19 mice. **a**, **b** High magnification representative photomicrographs of hippocampal sections stained for Acetyl Tau K280. Dotted lines depict CA1 area analyzed. **c**, **d** Quantitative analysis of Acetyl Tau K280 area and intensity in CA1 of the hippocampus. **e**, **f** Low and high magnification representative photomicrographs of hippocampal sections stained for AT8 and AT100. **g** Quantitative analysis of AT8 + cell count in CA1 of the hippocampus. **h** Quantitative analysis of AT100 + cell count in CA1 of the hippocampus. Box plots: center line represents the median, the limits represent the 25th and 75th percentile, and the whiskers represent the minimum and maximum values of the distribution. *p < 0.05; **p < 0.01

To elucidate the mechanism by which the AAV-Rbbp7 protected CA1 hippocampal neurons in PS19 mice, we first stained tissue with antibodies against Acetyl Tau K280, which measures tau acetylation (*n* = 6 mice/group). Notably, tau acetylation at lysine 280 has been shown to result in the hyper-phosphorylation of tau which impairs normal tau function thereby promoting tau secretion and propagation ([14, 15, 32]; PS19 mice show prominent Acetyl Tau K280 staining [15]). Quantitative analysis of Acetyl Tau K280 revealed a significant difference for area (*U* = 3, *p* = 0.0152; Fig. 5a–c) and intensity (*U* = 4, *p* = 0.0260; Fig. 5a, b, d), where the AAV-Rbbp7 injected mice show less tau acetylation than the PS19 AAV-GFP mice. Next, we stained tissue with antibodies against AT8 and AT100, which measure tau phosphorylation at Ser202/Thr205 and Thr212/Ser214, respectively (*n* = 6 mice/group). PS19 mice show prominent AT8 staining compared to other tau phosphorylation epitopes [1, 52]. Quantitative analysis of AT8 revealed a significant difference (*t*_(10)_ = 3.928, *p* = 0.0050; Fig. 5e, g) where AAV-Rbbp7 injected mice show less AT8 + cells than AAV-GFP mice. Next, quantitative analysis of AT100 + cell count in CA1 revealed a significant difference (*t*_(10)_ = 3.928, *p* = 0.0306; Fig. 5f, h) where PS19 AAV-Rbbp7 mice show a reduced AT100 + cell count than the PS19 AAV-GFP mice. Collectively, these results show that the levels of acetylated and phosphorylated tau are reduced in PS19 AAV-Rbbp7 mice, which help explain the reductions observed in neuronal loss given evidence showing that these markers are associated with tau-induced cell death [15, 52].

### p300 expression is negatively associated with Rbbp7 in PS19 mice and human MTG neurons

**Fig. 6:**
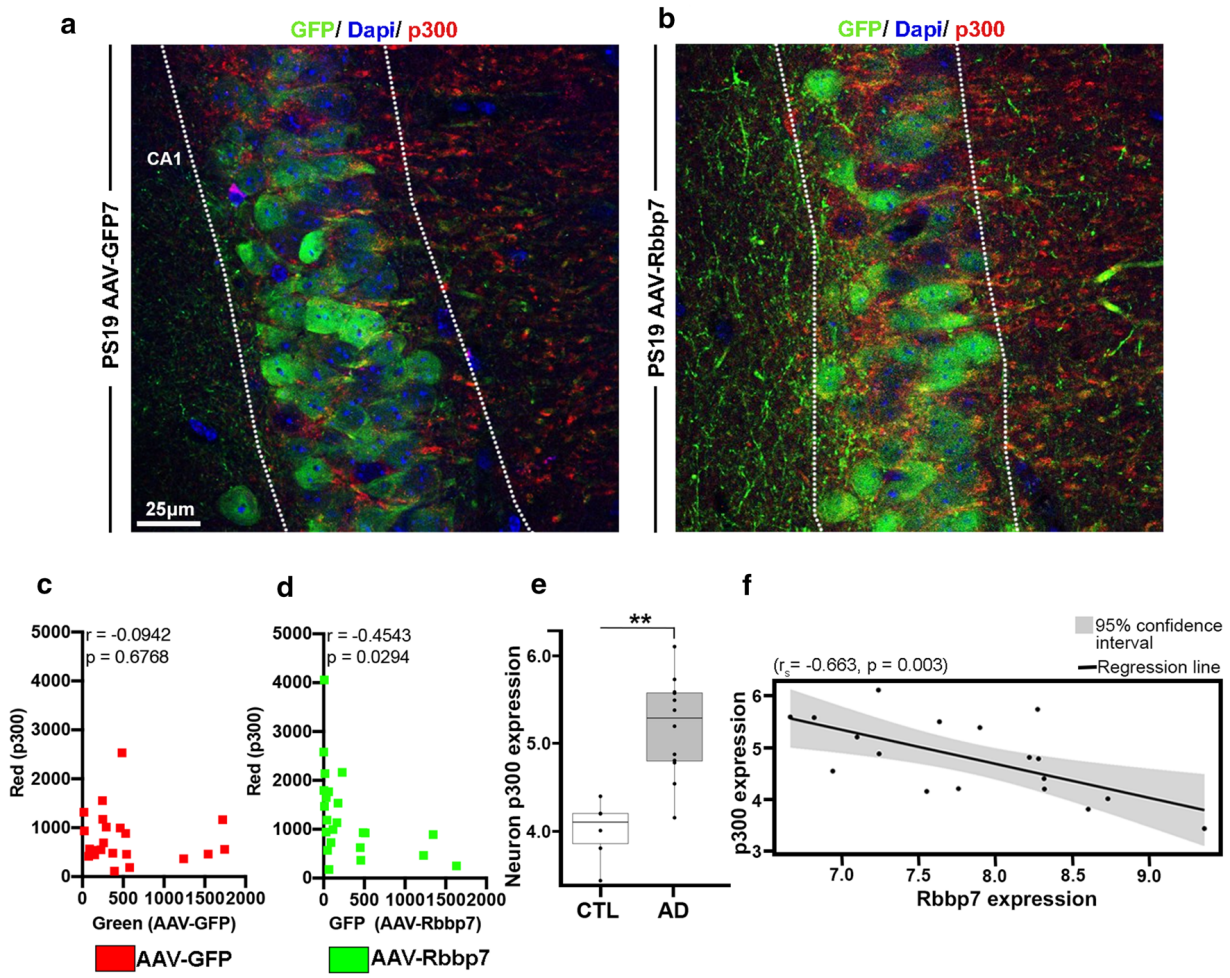
p300 expression is negatively associated with Rbbp7 in PS19 mice and human MTG neurons. **a**, **b** High magnification representative photomicrographs of hippocampal sections stained for GFP and p300. **c**, **d** Linear correlation of GFP and p300 in CA1 of the hippocampus for the PS19 AAV-GFP and PS19 AAV-Rbbp7 mice. **e** p300 mRNA expression is significantly elevated in laser-captured neurons from the MTG of AD patients. **f** Neuronal p300 mRNA expression is negatively correlated to Rbbp7 mRNA expression. Box plots: center line represents the median, the limits represent the 25th and 75th percentile, and the whiskers represent the minimum and maximum values of the distribution. **p < 0.01

Lastly, to determine whether the AAV-Rbbp7 alters p300 levels in PS19 mice, we co-stained tissue with antibodies against the GFP reporter and p300 (*n* = 5 mice/group). Notably, p300 increases the acetylation of tau at lysine 280 and Rbbp7 has been shown to bind to chromatin remodeling factors, including p300 [54]. Given that Rbbp7 interacts with p300, it may play a vital role in regulating tau acetylation and subsequent tau pathogenesis. We ran a linear correlation to determine whether the area stained for p300 within the CA1 was altered as a result of GFP expression in the AAVs. We found no significant correlation between GFP and p300 for the PS19 AAV-GFP mice (*r* = − 0.0942, *p* = 0.6768; Fig. 6a, c). We did however find a significant negative correlation in the PS19 AAV-Rbbp7 mice (*r* = − 0.4543, *p* = 0.0294; Fig. 6b, d), where the levels of p300 go down as the AAV-Rbbp7 expression goes up. To further validate the link between p300 and Rbbp7, we analyzed mRNA derived from MTG laser-capture neurons of AD (*n* = 6) and CTL (*n* = 12) patients. We found significantly higher neuronal p300 mRNA expression in AD patients compared to CTL (Log_2_ Fold Change = 1.242, FDR adj-*p* = 0.0015; Fig. 6e). Lastly, we ran a correlation analysis between Rbbp7 and p300 mRNA of MTG neurons and found a significant negative correlation (*r*_s_ = − 0.663, *p* = 0.003; Fig. 6f) illustrating that as Rbbp7 decreases, p300 increases. Collectively, these results highlight that p300 is elevated in MTG neurons of patients with AD and illustrate that increased expression of the AAV-Rbbp7 reduces p300 levels, which help to explain the observed reductions in tau acetylation and phosphorylation.

## Discussion

Our results show that Rbbp7 is downregulated in post-mortem brain tissue, specifically the middle temporal gyrus (MTG), of AD patients and is negatively associated with CERAD neuritic plaque density and Braak stage, but not with Lewy body stage. Additionally, we report a positive correlation between Rbbp7 and brain weight in post-mortem brains, suggesting that reduced Rbbp7 may account for brain atrophy seen in AD [10]. Interestingly, we find a similar effect in the 3xTg-AD and PS19 mouse models of AD possessing mutations leading to increased tau pathology [9, 38, 52]. Notably, APP/PS1 mice show comparable levels of Rbbp7 to NonTg, highlighting an association between Rbbp7 and pathogenic tau. We also identify a reduction of Rbbp7 in PS19 mice that precedes tau pathology and decreases significantly with age. We show that HT-22 cells and primary cortical neurons transfected with pathological TauP301L are more susceptible to cell death, and Rbbp7 co-transfection protects them from demise. Our results show that overexpression of Rbbp7 in the CA1 region of the hippocampus, an area heavily affected by tau acetylation, phosphorylation and neuronal loss, rescues these deficits in the PS19 mice [1, 52]. Lastly, we demonstrate that as the expression of Rbbp7 increases via the AAV-Rbbp7, the levels of p300 are reduced, which is consistent with correlations between Rbbp7 and p300 mRNA expression in human MTG neurons. Collectively, our results illustrate a novel role of Rbbp7 in protecting against tau pathology, which has significant therapeutic implications for AD and related tauopathies.

Interestingly, our results show that Rbbp7 is capable of blocking TNFα-induced cell death in primary cortical neurons. Studies have highlighted TNFα as an inducer of cell death [40]. Additionally, elevated TNFα has been shown to increase the presence of AD neuropathology in both human and mouse models of AD [16, 36]. However, whether TNFα is the mechanism by which pathological tau induces cell death remains to be known. Future work will determine if tau-induced cell death is mediated by TNFα, and if Rbbp7 disrupts this mechanism.

While we did observe genotype differences in the MWM task, delivery of the AAV-Rbbp7 into CA1 of the hippocampus did not rescue spatial learning and memory deficits in the PS19 mice. These results may be due to the specific targeting of the CA1 region. Previous reports have shown that the CA3 region of the hippocampus is involved in encoding spatial information during the MWM task [41]. Additionally, the entorhinal cortex, which is the major cortical input into the hippocampus, is required for spatial memory [43]. Given that we slowed the accumulation of tau and neuronal loss solely in CA1, tau burden is still likely accelerated in other hippocampal subregions and the entorhinal cortex, which may have confounded any AAV-Rbbp7 rescue effects. Future work will examine if increasing Rbbp7 globally improves cognition.

Our results show a reduction of tau acetylation at lysine 280 from increased expression of Rbbp7. Indeed, reports have shown that lysine 280 within the microtubule is the major binding site for tau acetylation [15]. Previous work has shown that tau acetylation inhibits normal tau function by impairing tau-microtubule interactions and promoting the aggregation of pathological tau [15]. More recently, work has shown that tau can be acetylated by p300 [14], and p300 has been shown to be chaperoned by Rbbp7 in a separate report [54]. p300 increased expression has been shown to acetylate tau at lysine 280, making it more pathogenic and prone to phosphorylation [34]. Our results show that overexpression of Rbbp7 via the AAV decreases p300 levels. Additionally, in human MTG neurons, increased Rbbp7 mRNA expression correlates with reduced p300 mRNA expression. Thus, it is tempting to speculate that Rbbp7 negatively regulates the expression of p300, thereby reducing tau acetylation. Notably, we found that increasing Rbbp7 levels reduced both tau acetylation and phosphorylation of AT8, which is consistent with previous reports examining the effects of p300 inhibition [14]. Collectively, these results highlight a novel role of Rbbp7 in tau acetylation and phosphorylation.

We found that the AAV-Rbbp7 significantly rescued neuronal loss in PS19 mice. Protection of neurons was confined to CA1 of the hippocampus, which is the anatomical location where the AAV-Rbbp7 was delivered and expressed. Notably, the AT8 epitope has been seen shown to be highly associated with neuronal loss in PS19 mice [1, 52]. Additionally, a very recent report showed that inhibiting the levels of p300 reduced tau acetylation at lysine 280 and resulted in a reduction of neuronal loss in the PS19 mice [14]. Moreover, inhibition of p300-induced tau acetylation has been shown to enhance tau turnover, suggesting that acetylation of tau inhibits proper degradation of pathogenic tau protein leading to accumulation of tau and cell death [34]. Interestingly, these results align with our human post-mortem data showing a negative correlation between Rbbp7 and Braak stage in AD. Additionally, our human data show a reduction in brain weight when Rbbp7 expression is reduced, suggesting that neuronal loss may account for the reduction in weight [10]. Thus, reductions in the expression of Rbbp7 may lead to increased tau acetylation, phosphorylation, and ultimately neuronal loss, thereby resulting in reductions of brain weight due to atrophy. Future work will seek to determine if global upregulation of Rbbp7 in PS19 mice reduces tau pathogenesis throughout the brain and improves cognitive deficits, as well as elucidate the specific interactions between Rbbp7, p300, and other modulators of acetylation.

In conclusion, our work identifies a novel target, Rbbp7, as being downregulated in AD post-mortem brain tissue, which is strongly associated with hallmark AD neuropathologies and brain weight. We also observed Rbbp7 dysregulation in neurons. Notably, Rbbp7 was strongly associated with tau pathology, as evidenced by Rbbp7 downregulation in mouse models of AD with tau pathology. Rbbp7 overexpression protects against cell death in immortalized hippocampal cells, primary cortical neurons and in the PS19 mouse model of tauopathy. This protection is likely due to reduced tau acetylation and phosphorylation resulting from Rbbp7’s negative regulation of p300. Collectively, our results highlight a novel role of Rbbp7 and set the stage for the development of future therapies to ameliorate tau pathology associated with tau acetylation in AD and related tauopathies.

## Supplementary Information

Below is the link to the electronic supplementary material.

Supplementary file1 (TIF 5555 KB) AAVs into CA1 of the hippocampus infects neurons. (a, b) Low and high magnification hippocampal sections stained against neuronal marker NeuN and the GFP reporter.
