# Supplementary figures and images for: Identification of retinoblastoma binding protein 7 (Rbbp7) as a mediator against tau acetylation and subsequent neuronal loss in Alzheimer’s disease and related tauopathies
Source: Acta Neuropathol. 2021 May 12;142(2):279–94. doi: 10.1007/s00401-021-02323-1 (PMC8270842; doi:10.1007/s00401-021-02323-1)

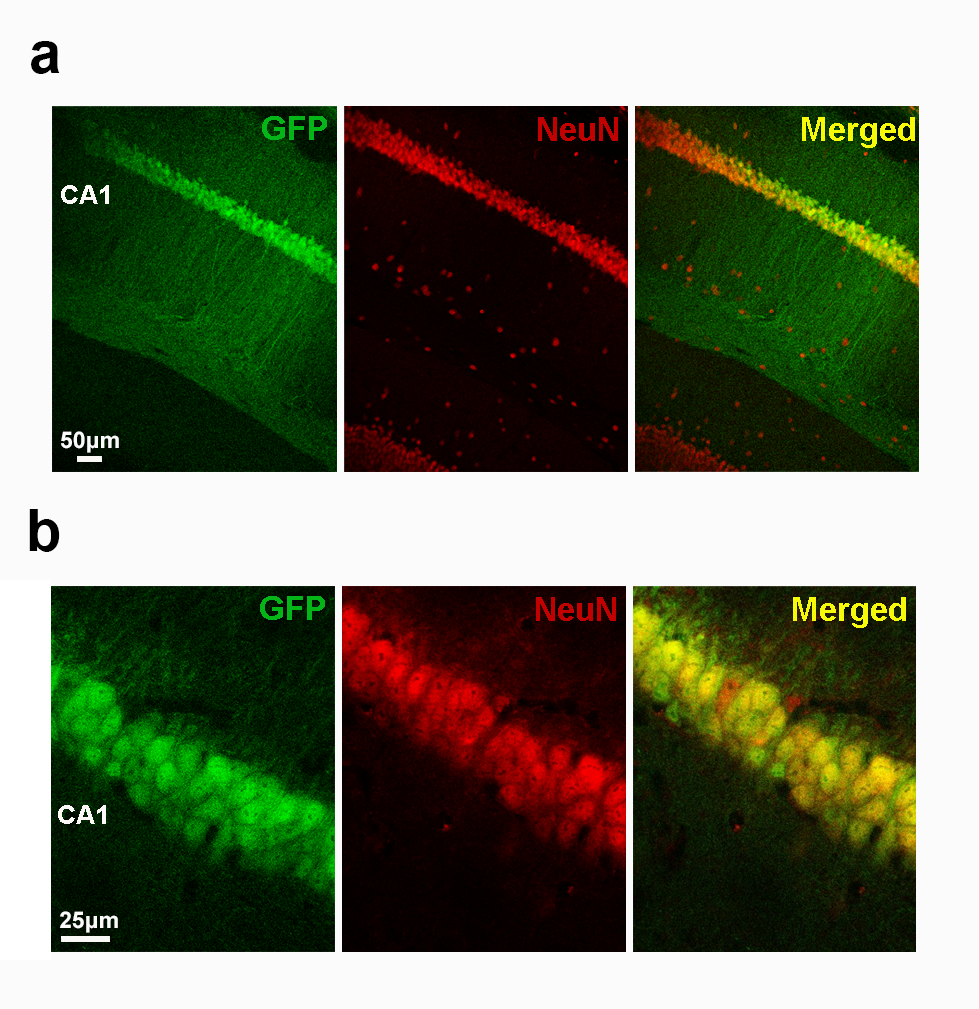

Supplement: Supplementary file 1 — Supplementary file1 (TIF 5555 KB) AAVs into CA1 of the hippocampus infects neurons. (a, b) Low and high magnification hippocampal sections stained against neuronal marker NeuN and the GFP reporter. [file 401_2021_2323_MOESM1_ESM.tif]
